# Sumoylation in p27kip1 via RanBP2 promotes cancer cell growth in cholangiocarcinoma cell line QBC939

**DOI:** 10.1186/s12867-017-0100-5

**Published:** 2017-09-07

**Authors:** Jun Yang, Yan Liu, Bing Wang, Hongzhen Lan, Ying Liu, Fei Chen, Ju Zhang, Jian Luo

**Affiliations:** 1 Department of Vascular Surgery, Tongji Hospital, Tongji Medical College, Huazhong University of Science and Technology, Wuhan, 430030 China; 2 Department of Geriatrics, Tongji Hospital, Tongji Medical College, Huazhong University of Science and Technology, 1095 Jiefang Avenue, Wuhan, 430030 Hubei People’s Republic of China; 3 Department of Bile Duct and Pancreatic Surgery, Tongji Hospital, Tongji Medical College, Huazhong University of Science and Technology, Wuhan, 430030 China; 4 CAS Key Laboratory of Genome Sciences & Information, Beijing Institute of Genomics, Chinese Academy of Sciences, Beijing, 100101 China; 5 Collaborative Innovation Center for Genetics and Development, Chinese Academy of Sciences, Beijing, 100101 China

**Keywords:** Sumoylation, p27kip1, RanBP2, Cholangiocarcinoma

## Abstract

**Background:**

Cholangiocarcinoma is one of the deadly disease with poor 5-year survival and poor response to conventional therapies. Previously, we found that p27kip1 nuclear-cytoplasmic translocation confers proliferation potential to cholangiocarcinoma cell line QBC939 and this process is mediated by crm-1. However, no other post-transcriptional regulation was found in this process including sumoylation in cholangiocarcinoma.

**Results:**

In this study, we explored the role of sumoylation in the nuclear-cytoplasmic translocation of p27kip1 and its involvement of QBC939 cells’ proliferation. First, we identified K73 as the sumoylation site in p27kip1. By utilizing plasmid flag-p27kip1, HA-RanBP2, GST-RanBP2 and His-p27kip1 and immunoprecipitation assay, we validated that p27kip1 can serve as the sumoylation target of RanBP2 in QBC939. Furthermore, we confirmed crm-1’s role in promoting nuclear-cytoplasmic translocation of p27kip1 and found that RanBP2’s function relies on crm-1. However, K73R mutated p27kip1 can’t be identified by crm-1 or RanBP2 in p27kip1 translocation process, suggesting sumoylation of p27kip1 via K73 site is necessary in this process by RanBP2 and crm-1. Phenotypically, the overexpression of either RanBP2 or crm-1 can partially rescue the anti-proliferative effect brought by p27kip1 overexpression in both the MTS and EdU assay. For the first time, we identified and validated the K73 sumoylation site in p27kip1, which is critical to RanBP2 and crm-1 in p27kip1 nuclear-cytoplasmic translocation process.

**Conclusion:**

Taken together, targeted inhibition of sumoylation of p27kip1 may serve as a potentially potent therapeutic target in the eradication of cholangiocarcinoma development and relapses.

**Electronic supplementary material:**

The online version of this article (doi:10.1186/s12867-017-0100-5) contains supplementary material, which is available to authorized users.

## Background

Cholangiocarcinoma, or bile duct cancer, is a gastrointestinal cancer with quite limited response to surgery treatment and other conventional therapies with poor long-term outcome [1–3]. Early diagnosis is difficult due to its anatomical and biological characteristics. Therefore, identifying molecular markers and mechanisms that underlie the carcinogenesis in cholangiocarcinoma is critical to the development of diagnostic tools in the early stage, novel potent drugs and effective therapies [1–6].

Sumoylation mainly functions at post-translational level affecting lots of cellular functions [7, 8] including nuclear-cytoplasmic transportation, transcription, protein stabilization, stress related processes and cell cycle regulation. It is mainly mediated by SUMO proteins, which is a group of small proteins that can covalently link or unlink to their target proteins to modify their functions [9]. Their function is quite similar to ubiquitin and the whole process is also analogous to the cascade reaction of ubiquitination.

RAN binding protein 2, or RanBP2 is a protein that belong to nucleoporin family that constitutes the nuclear pore complex [10]. RanBP2 has multiple domains, each of which can interact with various kinds of proteins such as importin-β [11], exportin-1/CRM1 [12], cox11 [13], the kinesin-1 [14] and KIF5C [15]. Particularly, exportin-1/CRM1 can utilize the zinc finger cluster domain of RanBP2 as the docking site [12]. By means of these domains, RanBP2 actively participates the protein shuttling program through the nucleomembrane. Cooperatively, there are multiple interactive proteins that help RanBP2 form complex on the nucleus membrane. One of them is the E2 enzyme UBC9 [16]. RanBP2 can promote SUMO1 transfer from UBC9 to the SUMO1 target SP100 [16]. Another protein partner is RanGAP which is the GTPase activating protein for Ran [17]. SUMO-RanGAP interacts with a domain at the carboxyl terminus of RanBP2. Evidence shows that sumoylation at the cytoplasmic filaments of the nuclear pore complex is one of the important activities on the nucleus membrane [18] and suggests that posttranslational sumoylation and nuclear import are tightly connected.

p27kip1, or cyclin-dependent kinase inhibitor 1B, is an enzyme inhibitor that can inhibit the activation of cyclin E-CDK2 [19] or cyclin D-CDK4 [20] complexes via binding to these proteins. It mainly helps stop the cell cycle progression at stage G1 [21]. Our previous study proved that crm-1 is enhanced in cholangiocarcinoma and can promote nuclear-cytoplasmic translocation of p27kip1 [22]. Interestingly, p53, a canonical anti-oncogene, was also found to be regulated by CRM-1 in the nucleocytoplasmic translocation and this process is modulated by sumoylation [23]. Also, we found p27kip1 mainly accumulates in the cytoplasm of cholangiocarcinoma cells and acts as a tumor suppressor. However, it is still elusive how this protein is transported to the cytoplasm and the possible epigenetic modification processes as sumoylation contributing in this process. Indeed, only Baldassarre and Schiappacassi et al. reported that K134 of p27kip1 is responsible for its sumoylation before it is transported through nuclear membrane in 293T/17 and Hela cells in response to TGF-β [24]. What’s happened of p27kip1 in the QBC939 and the role of sumoylation in this process is still unknown. Therefore, in this study, we investigated the possible sumoylation sites of p27kip1 and its possible regulatory proteins via immunoprecipitation, GST-pull down assays, MTS assay and EdU assay to evaluate the involvement of sumoylation in p27kip1, together with its clinical significance in cholangiocarcinoma.

## Methods

### Antibodies, plasmids and reagents

p27kip1 (ZA-0557) mouse anti-human monoclonal antibody, horseradish peroxidase (HRP)-labeled goat-anti-mouse IgG (ZB-2301), streptavidin–peroxidase immunostaining kit and 3,3′-Diaminobenzidine (DAB) were purchased from Beijing Zhongshan Co. (Beijing, China); rabbit anti-CRM-1 (H-300; sc-5595); SUMO-1 (sc-5308) mouse anti-human monoclonal antibody, His-probe (sc-804) rabbit polyclonal antibody, HA-probe (sc-7392) mouse monoclonal antibody and histone H3 (sc-10809) rabbit polyclonal antibody, CDK-2 (sc-6248) mouse anti-human antibody, p-CDK2 (sc-101656) rabbit anti-human antibody were purchased from Santa Cruz Biotechnology (Santa Cruz, CA, USA). Anti-DDDDK tag (or anti-flag tag) (ab1162) rabbit polyclonal antibody was purchased from Abcam (Abcam, MA, USA). Glutathione Agarose (cat#16100) was purchased from Pierce ThermoFisher Scientific Corp. (ThermoFisher, USA). The ECL reagents were purchased from Pierce Chemical Co. (Rockford, IL, USA). Two pair of siRNAs targeting CRM-1 were purchased from Ribobio Corp. (Guangzhou, China) and sequence are listed as follows, siRNA1: GAAGUACUGACACAUUUAA; siRNA2: GGCUGCUGAACUCUAUAGA.

Plasmids for flag-p27kip1-wt, flag-p27kip1-K73R, HA-ranBP2, GST-RanBP2, flag-Crm1 and His-p27kip1 were purchased from Ribobio Corp. (Guangzhou, China).

### Cytoplasmic and nuclear protein isolation

The cytoplasmic and nuclear proteins were isolated with the ProteoJET Cytoplasmic and Nuclear Protein Extraction Kit (Fermentas Corp.; Burlington, ON, Canada) by following the manufacturer’s instructions. Tissue samples were gently homogenized in PBS with protease inhibitors after pre-cooled PBS rinsing. The solution was then centrifuged at 250*g*, 5 min at 4 °C. The supernatant was discarded and 500 μl of cell lysis buffer containing protease inhibitors and DTT was added to 100 mg of tissue, mixed gently by vortexing, and incubated in ice for 10 min. The cytoplasmic fraction was isolated first via centrifugation at 500*g*, 7 min at 4 °C. The nuclei pellet was set on ice and the supernatant was centrifuged at 20,000*g*, 15 min at 4 °C to clear the cytoplasmic protein extract. Then it was transferred to a new tube for analysis. At the same time, the nuclei pellet was washed in 500 μl nuclei washing buffer with protease inhibitors and DTT by vortexing briefly, and then incubate in ice for 2 min. After centrifugation at 500*g*, 7 min at 4 °C, the resultant supernatant was removed. After repeating this wash for another 1–2 times, the volume of the nuclei pellet was estimated and 10 volumes of ice-cold nuclei storage buffer containing protease inhibitors and DTT was used for resuspension. The suspension was transferred to a new tube for centrifugation at 20,000*g*, 5 min, 150 μl of ice-cold nuclei storage buffer with protease inhibitors and DTT was added to the nuclei pellet. The nuclei were lysed with one tenth of the volume of nuclei lysis reagent. After short vortexing and incubation on a rotating bed (900–1200 rpm), 15 min at 4 °C, the resultant nuclear lysate was processed with centrifugation at 20,000*g*, 5 min at 4 °C, and the supernatant containing the nuclear protein extract was transferred to a new tube for analysis.

### GST-pull down and co-immunoprecipitation assays

GST-pull down was undertaken as mentioned by Sambrook [25]. To investigate protein interactions, co-immunoprecipitation was performed in QBC939 cells. The cells were transfected with different vectors and lysed in NETN buffer. Specific antibody and Protein G Agarose (Roche) were incubated with the cell lysate overnight at 4 °C. The resins were washed four times with buffer NETN. After elution by 1× loading buffer, heated at 95 °C for 5 min, the bound proteins were further analyzed with western blotting.

### Western blot analysis

Equal amounts of nuclear or cytoplasmic proteins were examined by SDS-PAGE electrophoresis. The proteins were transferred to a PVDF membrane and blocked in blocking buffer (20% skim milk in TBST) for 2 h at 25 °C. The membrane was incubated at 25 °C for 1 h with primary antibodies against SUMO1 (1:1000); flag (1:1000); HA (1:1000); p-CDK2 (Thr 160) (1:1000); CDK-2 (1:2000); His (1:1000); p27kip1 (1:2000); CRM-1 (1:1000); and (1:1000); After further incubation at 4 °C overnight, the membranes were incubated with HRP-conjugated secondary antibodies (1:1000) for 2 h at room temperature. The membrane was processed with electrochemical luminescence (ECL) and exposed to X-ray film. The expression level were quantified with a grayscale scanner. H3 (1:2000) was included as loading controls.

### Cell viability assay

QBC939 cells were lentiviral infected with flag-p27kip1 and/or HA-RanBP2, HA-Crm1 vector after lentivirus packaging for approximately 24 h. After the treatment, the above two groups together with no treatment QBC939 cells were transferred to 96-well plate with the density of 1000 cells per well. Cell viability was evaluated by MTS assay (Promega, USA) and EdU assay (Ribo, Guangzhou, China) according to manufacturer’s protocol.

### In vitro sumoylation assay

This assay was undertaken as described by Lovista et al. [24]. The whole reaction was undertaken in 30 μl reaction system with 50 mM Tris–HCl (pH 7.4), 150 mM NaCl, 5 mM MgCl_2_, 2 mM DTT, 2 mM ATP, 270 ng SAE1/SAE2 (Boston Biochem, USA), 300 ng UBC9 (Boston Biochem, USA), 4 µg SUMO1 (Boston Biochem, USA), and 3 µg His-p27 and incubated for 2 h at 30 °C. Then all samples were sent for western blotting analysis.

### Statistical analysis

All experiments were repeated three times. Statistical analyses were carried out using SPSS software v. 20.0 (SPSS Inc.; Chicago, IL, USA). Fisher’s exact test was used to determine significant differences between groups of data; Chi square (χ^2^) test was used to compare protein expression percentage, Spearman’s Rank Correlation test was used to compare pairs of variables. All values are shown as mean ± SEM unless otherwise indicated and one way ANOVA was adopted to examine the difference between the CRM-1 knock-down group, blank group and vector group. All statistical significant results were shown in *** for p < 0.001; ** for p < 0.01 and * for p < 0.05.

## Results

### p27kip1 can be sumoylated at K73 modification site

**Fig. 1:**
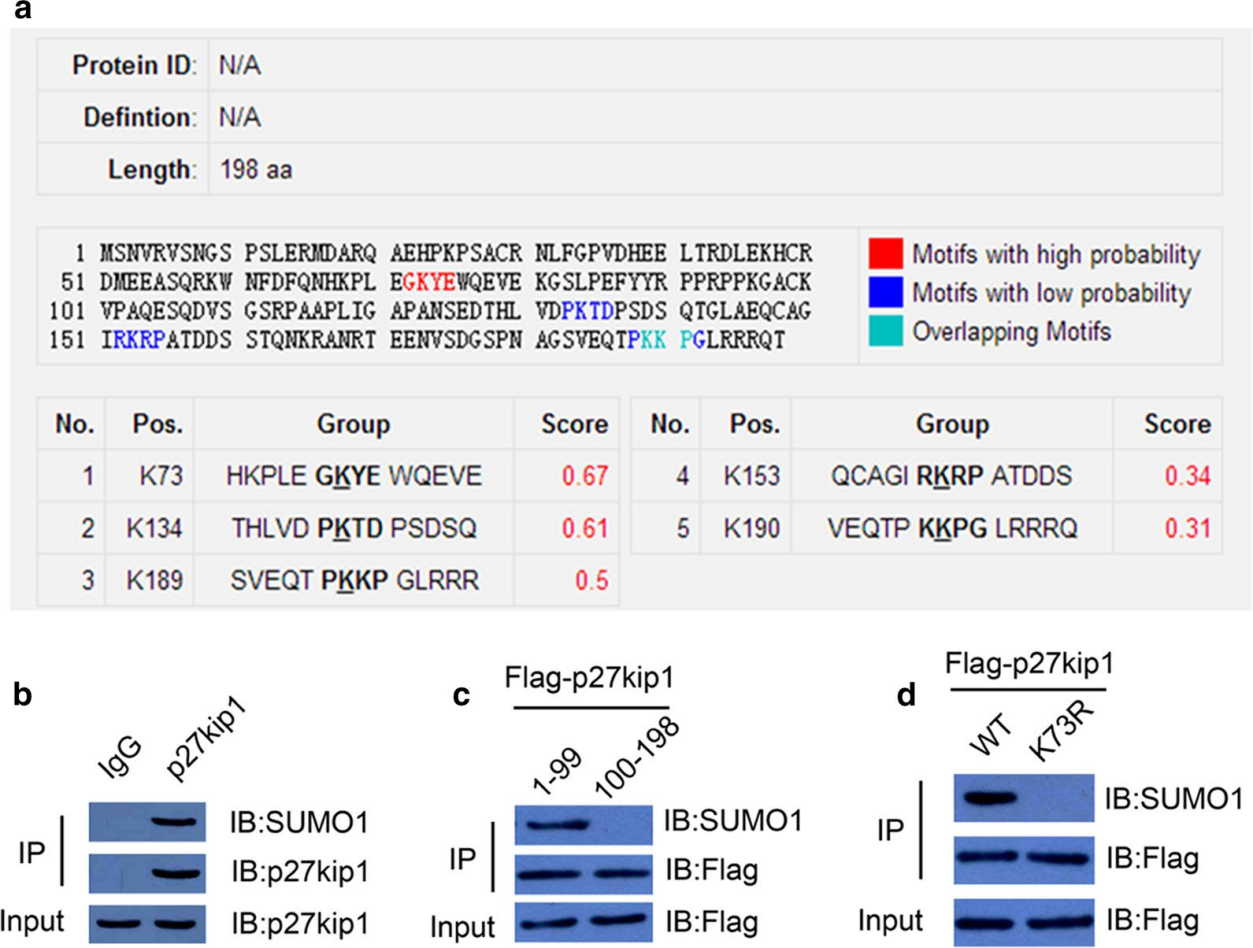
p27kip1 can be sumoylated at K73 modification site. **a** The sites predicted by the model developed by Xue and Ren at the website of http://sumosp.biocuckoo.org. **b** The immunoprecipitation result with the antibody of SUMO1 as the probe. Also, plasmids expressing 1st–99th amino acid (aa) and 100th–198th aa with or without mutation in potential sumoylation sites fused with flag tag were used for protein immunoprecipitation and purification in QBC939 cells, followed with anti-SUMO1 antibody examination, as shown in **c**. Also, flag-p27kip1-WT and K73R point mutated form of p27kip1 were constructed and transfected into QBC939 cells, immunoprecipitation with flag antibody reveals that K73 is the sumoylation site for p27kip1 (**d**)

As shown in Fig. 1a, there are five possible sumoylation sites for p27kip1 according to the model developed by Xue and Re [26, 27] at the website of http://sumosp.biocuckoo.org. In order to further validate the sumoylation site for p27kip1, immunoprecipitation were undertaken by using anti-p27kip1, anti-flag and anti-SUMO1 antibodies, together with flag-p27kip1-WT and K73R point mutated form of p27kip1 constructs. Results reveal that K73 is the sumoylation site for p27kip1, as shown in Fig. 1b–d.

### RanBP2 can increase the sumoylation of p27kip1

**Fig. 2:**
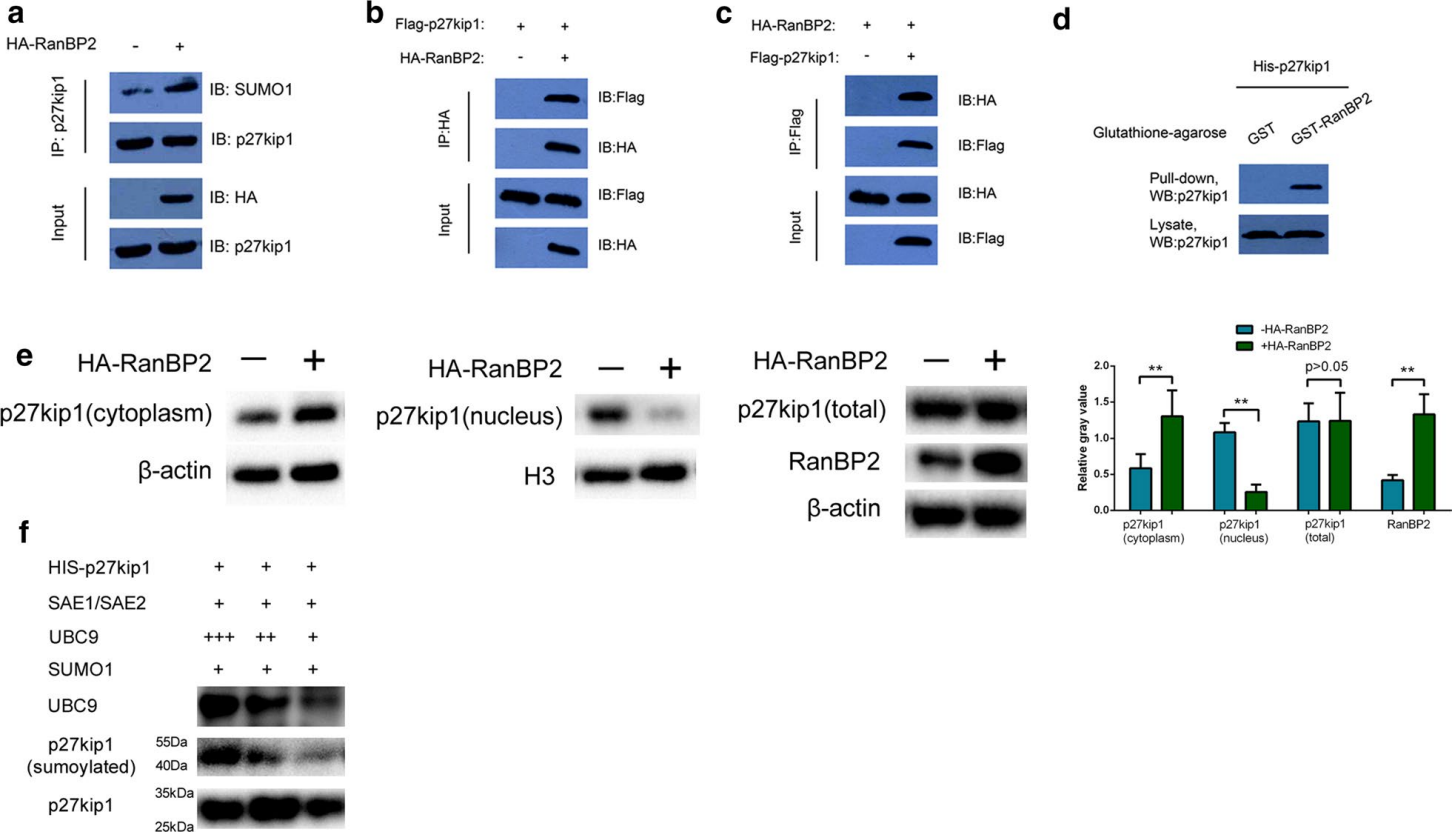
RanBP2 can increase the sumoylation of p27kip1. RanBP2-HA plasmid was constructed and transfected to QBC939 cells. Immuno-precipitation with anti-p27kip1 antibody verified that RanBP2 can increase the sumoylation of p27kip1 (**a**). Also, flag-p27kip1 plasmid was constructed and transfected into either QBC939-RanBP2-HA or QBC939-control cells. Immuno-precipitation with anti-HA antibody proved that p27kip1 can bind with RanBP2 (**b**). Correspondingly, HA-RanBP2 plasmid was transfected into either QBC939-flag-p27kip1 or QBC939-control cells. Immunoprecipitation result with anti-flag antibody proved that RanBP2 can bind with p27kip1. **c**. Moreover, GST-RanBP2 and His-p27kip1 plasmids were constructed for GST-pull down assay. As shown in **d**, p27kip1 proved to be one of the binding targets of RanBP2. **e** HA-RanBP2 plasmids and its control vector were transfected into QBC939 cells. Cytoplasmic proteins, nuclear proteins and total cellular proteins were separately extracted from the same batch of cells and sent for WB. Also, RanBP2 was validated to be overexpressed after HA-RanBP2 transfection. **f** In vitro sumoylation assay was undertaken by his-tagged p27kip1 proteins were incubated with SAE1/SAE2, SUMO1 and UBC9 in increasing doses (**p < 0.01)

RanBP2-HA plasmid was constructed and transfected to QBC939 cells. Immuno-precipitation with anti-p27kip1 antibody verified that RanBP2 can increase the sumoylation of p27kip1 (Fig. 2a). Also, flag-p27kip1 plasmid was constructed and transfected into either QBC939-RanBP2-HA or QBC939-control cells. Immuno-precipitation with anti-HA antibody proved that p27kip1 can bind with RanBP2 (Fig. 2b). Correspondingly, HA-RanBP2 plasmid was transfected into either QBC939-flag-p27kip1 or QBC939-control cells. Immunoprecipitation result with anti-flag antibody proved that RanBP2 can bind to p27kip1 (Fig. 2c). Moreover, GST-RanBP2 and His-p27kip1 plasmids were constructed for GST-pull down assay. As shown in Fig. 2d, p27kip1 proved to be one of the protein partners of RanBP2. Also, we analyzed the cytoplasmic and nuclear p27kip1 levels and found that p27kip1 in the nucleus is transported through nuclear membrane after RanBP2 is overexpressed (Fig. 2e). In order to further justify the sumoylation of p27kip1 in QBC939 cells, we did an in vitro sumoylation assay (Fig. 2f) and found p27kip1 can be modified via interaction with UBC9, SUMO1 and SAE1/SAE2.

### Sumoylation p27kip1 is indispensable for its nucleus-cytoplasmic translocation mediated by Crm-1

**Fig. 3:**
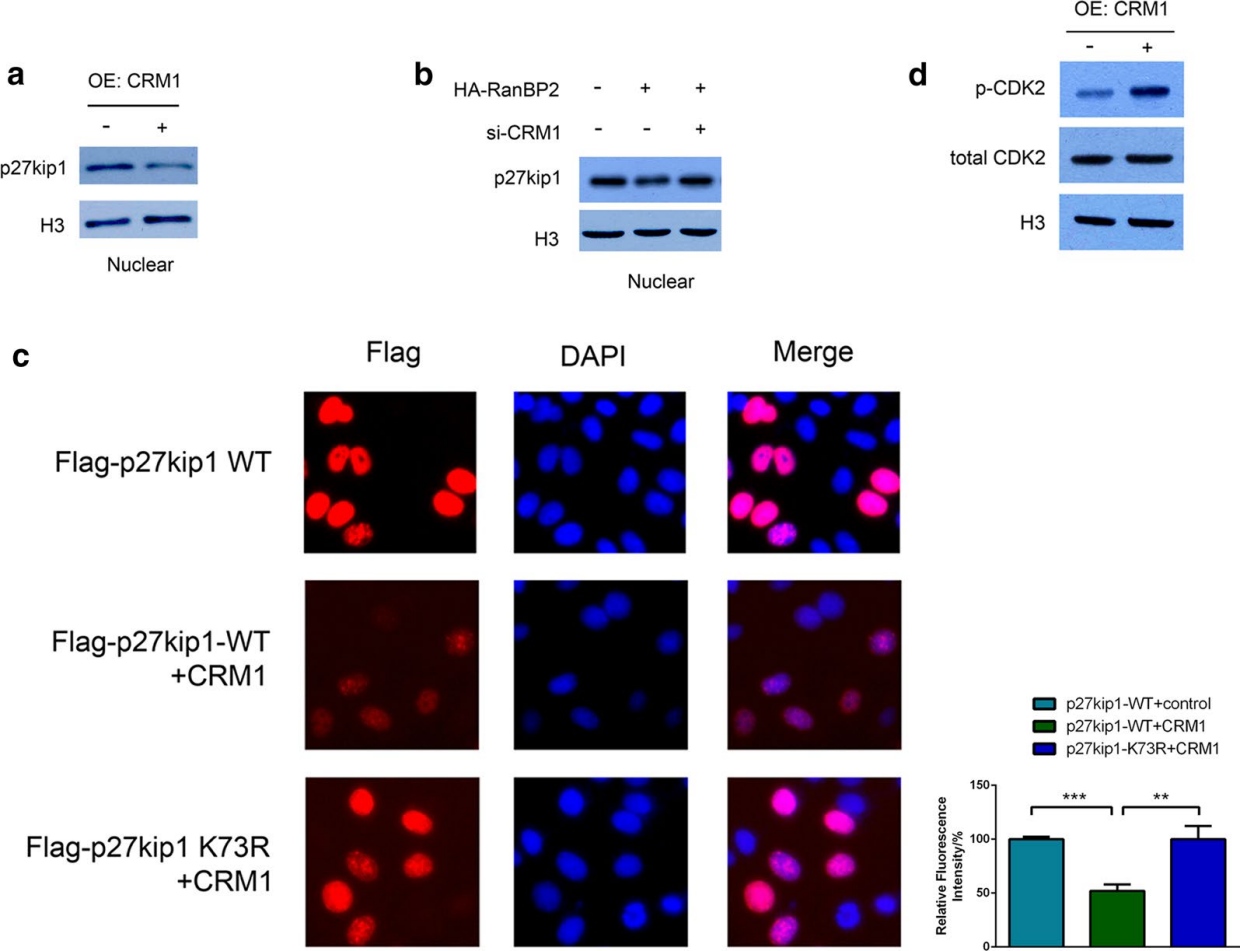
Sumoylation p27kip1 is indispensable for its nucleus-cytoplasmic translocation mediated by Crm-1. Nuclear and cytoplasmic protein were separately extracted. **a** shows that p27kip1 expression in the nucleus is reduced after overexpression (OE) of Crm-1 in QBC939 cells. Also, as we transfect the HA-RanBP2 plasmid into QBC939 cells, p27kip1 expression in the nucleus was decreased (**b**). Moreover, when we transfect QBC939-HA-RanBP2 cells with siRNA suppressing Crm-1, p27kip1 level was rescued in the nucleus (**b**). Additionally, immunofluorescence staining with anti-flag antibody also verifies that Crm-1 overexpression can suppress nuclear p27kip1 (**c**) while the level of a K73R point mutated form of p27kip1 in the nucleus cannot be changed by Crm-1 overexpression (**c**), in which the fluorescence intensity was determined by the red signal/blue signal in each cell’s nuclear area. Further, we found the phosphorylated form of CDK-2 in the nucleus was reduced when Crm-1 overexpressed in QBC939 cells (**d**)

Nuclear and cytoplasmic protein were separately extracted. Figure 3a shows that p27kip1 expression in the nucleus is reduced after overexpression of Crm-1 in QBC939 cells. Also, as we transfect the HA-RanBP2 plasmid into QBC939 cells, p27kip1 expression in the nucleus was decreased (Fig. 3b). Moreover, when we transfect QBC939-HA-RanBP2 cells with siRNA suppressing Crm-1, p27kip1 level was rescued in the nucleus (Fig. 3b; Additional file 1: Fig. S1). Additionally, immunofluorescence staining with anti-flag antibody also verifies that Crm-1 overexpression can suppress nuclear p27kip1 (Fig. 3c; Additional file 1: Fig. S2) while the level of a K73R point mutated form of p27kip1 in the nucleus cannot be changed by Crm-1 overexpression (Fig. 3c). Further, we found the phosphorylated form of CDK-2 in the nucleus was reduced when Crm-1 overexpressed in QBC939 cells (Fig. 3d).

### Sumoylation of p27kip1 promotes the proliferation of cholangiocarcinoma QBC939 cells

**Fig. 4:**
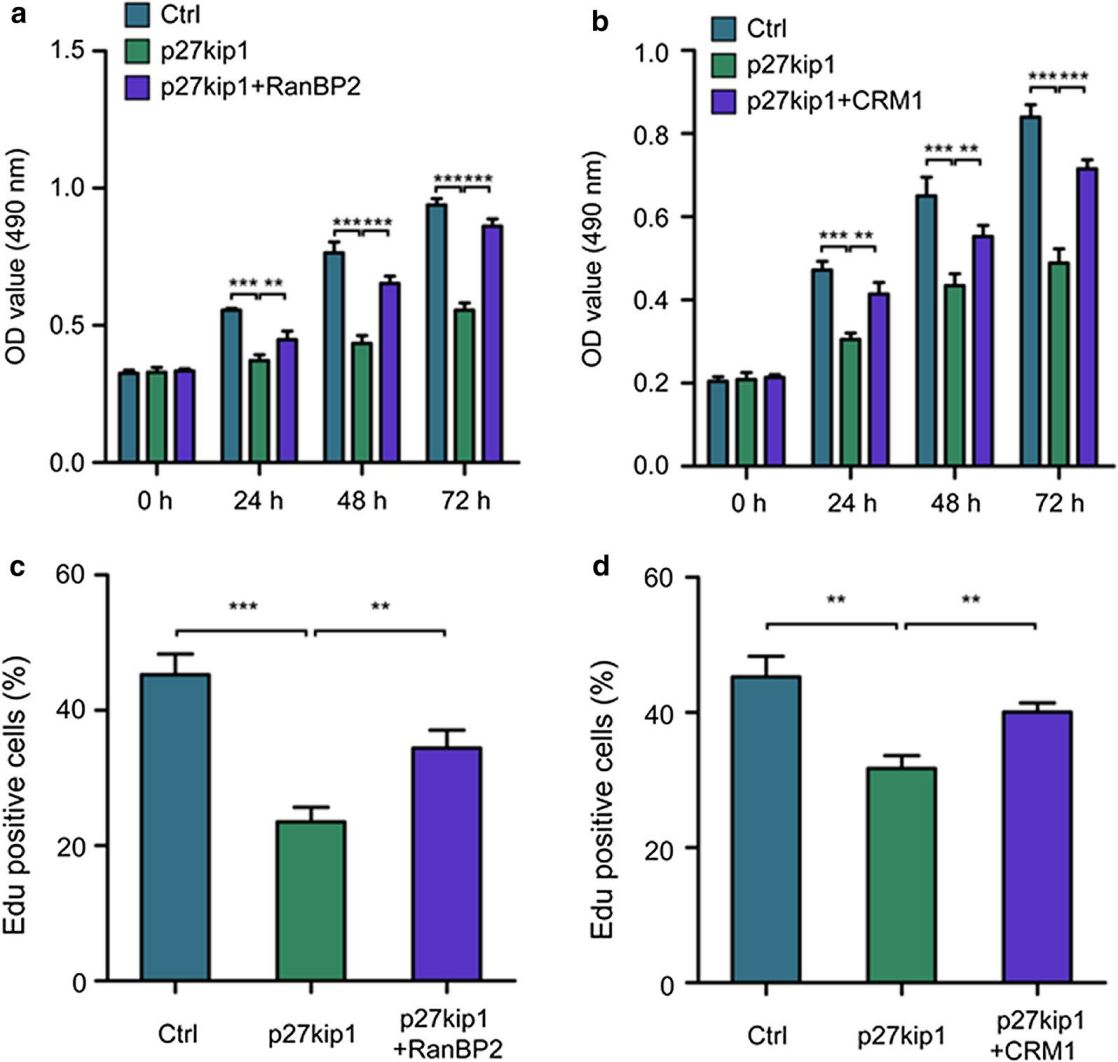
Sumoylation of p27kip1 promotes the proliferation of cholangiocarcinoma QBC939 cells. p27kip1 was found to be able to suppress the growth of QBC939 cells when it was artificially overexpressed in QBC939 cells (**a**). However, this effect can be partially rescued by overexpression of RanBP2 (**a**). Similar effect was observed when Crm-1 can partially rescue the anti-proliferative effect brought by p27kip1 overexpression (**b**). Moreover, EdU assay also verifies that both RanBP2 (**c**) and Crm-1 (**d**) can partially reverse the effect brought by overexpression of p27kip1 in QBC939 cells (**p < 0.01, ***p < 0.001)

p27kip1 was found to be able to suppress the growth of QBC939 cells when it was artificially overexpressed in QBC939 cells (Fig. 4a). However, this effect can be partially rescued by overexpression of RanBP2 (Fig. 4a). Similar effect was observed when Crm-1 can partially rescue the anti-proliferative effect brought by p27kip1 overexpression (Fig. 4b). Moreover, EdU assay also verifies that both RanBP2 (Fig. 4c) and Crm-1 (Fig. 4d) can partially reverse the effect brought by overexpression of p27kip1 in QBC939 cells (**p < 0.01, ***p < 0.001).

## Discussion

SUMO proteins were firstly discovered and validated over a decade ago, when a cellular protein as small as 12 kDa which bears 18% homology to the ubiquitin protein was found. There are four different genes coding for different SUMO proteins [28], including SUMO1, 2, 3 and 4. SUMO 2 and 3 shared 92% common sequences but only 48% identity as compared to SUMO1. Bellail et al. reported that SUMO1 modification can stabilize CDK6 protein and drive the cell cycle and glioblastoma progression [8]. Also, SUMO 1 is said to modify LKB1, which is a major upstream kinase of the energy sensor AMPK, enabling LKB1 to recognize and activate AMPK in energy–stress in cancer microenvironment [7]. However, there’s still a lack of knowledge in sumoylation of p27kip1 in cholangiocarcinoma. Spagnuolo et al. reported that reduction of sumoylation in S100A4 would decrease its nuclear import brought by low dose paclitaxel, leading to halts of tumor invasiveness and hematogenous metastatization [4]. Baldassarre and Schiappacassi et al. reported that K134 of p27kip1 is the sumoylation site in tumor-derived cell in response to TGF-β [24]. There are no other study focusing on the post-translational regulation in cholangiocarcinoma.

p27kip1 is a tumor suppressor gene that actively participate in regulating the cell cycle progression negatively at the G1/S checkpoint via inhibiting CDK2. As shown in Fig. 3d, CDK2 phosphorylation was remarkably enhanced after CRM-1 overexpression. This is consistent with our speculation that CRM-1 controls p27kip1 nucleocytoplasmic translocation thereby eliminating its inhibitory effect on CDK2 and promoting G1/S entry. Aberrant expression or localization of p27kip1 weakens its inhibitory effect in the cell cycle, leading to uncontrolled cell growth and carcinogenesis [29]. Downregulation of p27kip1 and abnormal subcellular localization have been reported in a variety of tumor types [30]. Our previous study shows that p27kip1 nuclear-cytoplasmic translocation also happens in the cholangiocarcinoma and this might due to the enhanced level of crm-1 in this cancer. The downregulation of crm-1 can remarkably reduce the cytoplasmic level of p27kip1, resulting in the improvement of cancer cell proliferation.

In malignancy transformation, p27kip1 is often silenced via synthesis impairment, degradation acceleration, or aberrant cellular localization as we study in the cholangiocarcinoma. P27kip1 silencing generally occur posttranscriptionally brought by the activation of pathways as receptor tyrosine kinases (RTKs), phosphatidylinositol 3-kinase (PI3K), Ras-mitogen activated protein kinase (MAPK). Enhancement of these pathways promoting p27kip1 proteolysis allows cholangiocarcinoma cells to divide and proliferate. Once p27kip1 is phosphorylated by Src at tyrosine 74 or 88, the inhibition to cyclinE-cdk2 would be unchained. Also, in our study, when p27kip1 is sumoylated or tagged by SUMO1 at K73, the transportation of this protein from nucleus to cytoplasm is accelerated with the aid of RanBP2 and crm-1.

Also, evidence shows that RanBP2 mainly functions as SUMO E3 ligase and facilitates protein import or export from the nuclear membrane. Packham et al. [31] reported that the sumoylation of IGF-1R would help its nuclear translocation by RanBP2 and importin-β, leading to the failure of anti-IGF-1R therapy in cancers. However, there are also studies finding that RanBP2 can work as a tumor suppressor gene via sumoylation of different target proteins as Topoisomerase II (TopoII) [32]. Considering our study in the cholangiocarcinoma, together with all the publications concerning RanBP2 and sumoylation, the role of RanBP2 is more likely dependent on its target protein. In our study, the target protein p27kip1 mainly acts as a tumor-suppressor gene in the nucleus, RanBP2 and SUMO1 act as oncogenes by promoting the nuclear-cytoplasmic translocation and debilitate the G1-arrest brought by p27kip1 accumulation in the nucleus. In spite of all the facts we have about the sumoylation regulation in p27kip1, there’re still much more to be discovered including the possible oncogenic effect of other SUMO proteins because of their overlapping and compensatory functions, how the regulatory network of SUMO1, RanBP2, Crm-1, p27kip1 and other cell cycle proteins collaborate in the maintenance of cholangiocarcinoma cells and etc. In spite of these challenges, our investigation in the sumoylation of p27kip1 by RanBP2 and Crm-1 sheds light on the regulation of cancer cell growth in cholangiocarcinoma and offers a novel therapeutic target for the treatment and eradication of this cancer in the future.

## Conclusion

According to the results we have, it’s promising that targeted inhibition of sumoylation of p27kip1 may serve as a potentially potent therapeutic target in the eradication of cholangiocarcinoma development and relapses.

## Additional file

**Additional file 1. Figure S1.** CRM-1 expression in the total cellular protein were validated to be downregulated after siRNA transfection. This is the validation result for CRM-1 knockdown mediated by siRNA. **Figure S2.** CRM-1 expression in the total cellular protein were validated to be upregulated after flag-CRM1 transfection. This is the validation result for CRM-1 artificial overexpression mediated by flag-CRM1 vector.

## Abbreviations

OE: overexpression
Crm-1: exportin 1
RanBP-1: RAN binding protein 1
