# Supplementary material for: Sumoylation in p27kip1 via RanBP2 promotes cancer cell growth in cholangiocarcinoma cell line QBC939
Source: BMC Mol Biol. 2017 Sep 7;18:23. doi: 10.1186/s12867-017-0100-5 (PMC5590128; doi:10.1186/s12867-017-0100-5)
Supplement: Supplementary file 1 — Additional file 1. Figure S1. CRM-1 expression in the total cellular protein were validated to be downregulated after siRNA transfection. This is the validation result for CRM-1 knockdown mediated by siRNA. Figure S2. CRM-1 expression in the total cellular protein were validated to be upregulated after flag-CRM1 transfection. This is the validation result for CRM-1 artificial overexpression mediated by flag-CRM1 vector. [file 12867_2017_100_MOESM1_ESM.pdf]

Supplementary Figure 1

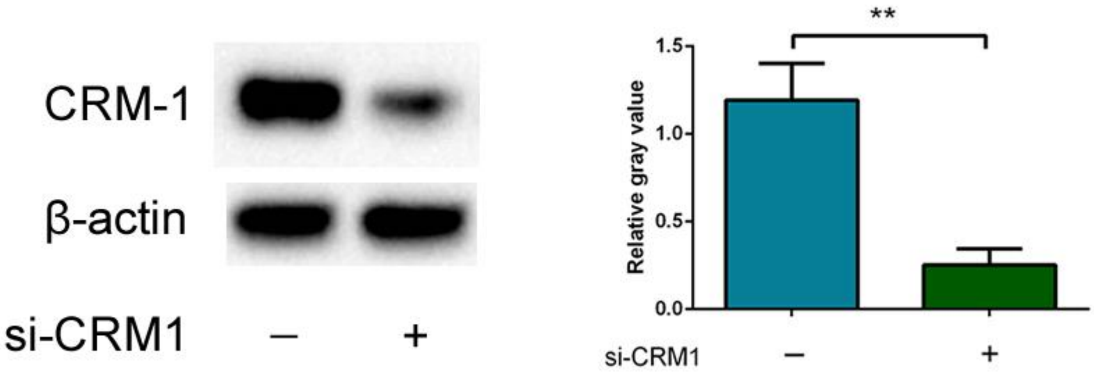

Supplementary Figure 1. CRM-1 expression in the total cellular protein were validated to be downregulated after siRNA transfection. siRNA1 and siRNA2 were utilized to transfect QBC939 cells and what's shown is result from siRNA1. The relative gray value of CRM-1 with respect to internal control protein β-actin were evaluated and statistically analyzed. (**: $p<0.01$)

Supplementary Figure 2

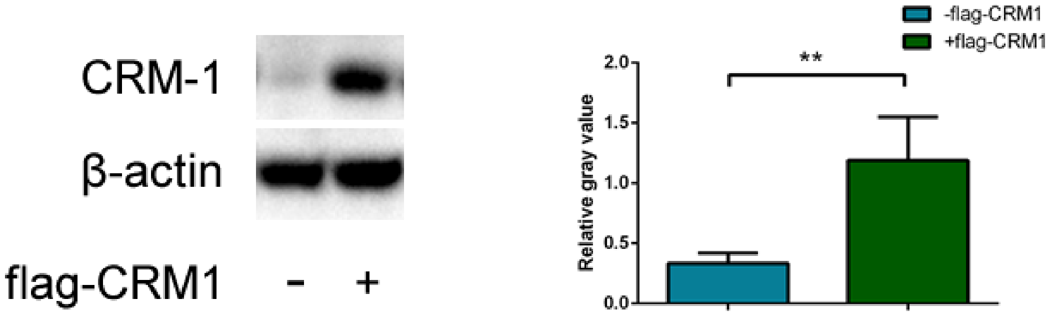

Supplementary Figure 2. CRM-1 expression in the total cellular protein were validated to be upregulated after flag-CRM1 transfection. Flag-CRM1 and its control vector was used to transfect QBC939 cells. The relative gray value of CRM-1 with respect to internal control protein β-actin was evaluated and statistically analyzed. (**: $p<0.01$)
